# Fanconi anemia protein FANCD2 inhibits TRF1 polyADP-ribosylation through tankyrase1-dependent manner

**DOI:** 10.1186/2041-9414-2-4

**Published:** 2011-02-12

**Authors:** Alex Lyakhovich, Maria Jose Ramirez, Andres Castellanos, Maria Castella, Amanda M Simons, Jeffrey D Parvin, Jordi Surralles

**Affiliations:** 1 Department of Genetics and Microbiology, Universitat Autonoma de Barcelona, Edifici C, Bellaterra, Barcelona 08193, Spain; 2 Cancer and Stem Cell Research Program, Duke-NUS Graduate Medical School, College Rd. 6, 169857, Singapore; 3 Centre for Biomedical Network Research on Rare Diseases (CIBERER), Instituto de Salud Carlos III, Spain; 4 Fred Hutchinson Cancer Research Center, Seattle, USA; 5 Department of Pathology, Harvard Medical School and Brigham and Women's Hospital, Boston, MA, USA; 6 Department of Biomedical Informatics, The Ohio State University, 460 W. 12th Avenue, Columbus, OH, USA

## Abstract

**Background:**

Fanconi anemia (FA) is a rare autosomal recessive syndrome characterized by developmental abnormalities, progressive bone marrow failure, and predisposition to cancer. The key FA protein FANCD2 crosstalks with members of DNA damage and repair pathways that also play a role at telomeres. Therefore, we investigated whether FANCD2 has a similar involvement at telomeres.

**Results:**

We reveal that FANCD2 may perform a novel function separate to the FANCD2/BRCA pathway. This function includes FANCD2 interaction with one of the telomere components, the PARP family member tankyrase-1. Moreover, FANCD2 inhibits tankyrase-1 activity in vitro. In turn, FANCD2 deficiency increases the polyADP-ribosylation of telomere binding factor TRF1.

**Conclusions:**

FANCD2 binding and inhibiting tankyrase-1PARsylation at telomeres may provide an additional step within the FA pathway for the regulation of genomic integrity.

## Background

Fanconi anemia (FA) is a rare recessive disorder associated with chromosomal fragility, aplastic anemia, congenital abnormalities and a predisposition to cancer [1,2]. Cells from FA patients exhibit hypersensitivity to DNA cross-linking agents suggesting the role of FA proteins in the repair of damaged DNA [3,4]. Currently, at least 14 FA genes are known to exist, each of them representing a different FA subtype [5-7]. Although they have very few similarities, the encoded FA proteins cooperate in a common FA/BRCA pathway by forming several complexes, where the activation of a key FA protein FANCD2 (and FANCI) seems to orchestrate the cascade of events in response to DNA damage [8,9].

FA proteins crosstalk with several proteins involved in both DNA damage response and telomere regulation [10-13]. Telomeres, the ends of chromosomes, consist of TTAGGG tandem repeats (in mammals) forming a T-loop structure and a 3' G-rich single-stranded overhang that invades the telomeric tracts forming a D loop [14-18]. In most human somatic cells telomeres undergo shortening with each cycle of cell division due to what is known as the "end-replication problem" [19,20]. To prevent such shortening, a specialized enzyme called telomerase serves to maintain telomere length [21]. In normally dividing somatic cells telomerase is insufficiently active to compensate for telomere shortening and telomeres undergo attrition with each round of cell division. In turn, telomeres in cancer cells are maintained either by the activation of telomerase or by the homologous recombination mechanism known as alternative lengthening of telomeres (ALT) [22-24].

Telomeres are capped by a complex of proteins called shelterin, which contains TRF1, TRF2, POT1 and associated proteins including TIN2, TPP1, Rap1 and poly-(ADP-ribose)-polymerase enzymes tankyrase 1 and 2 (TNKS1/2) [23,25]. TIN2 negatively regulates telomere length by changing the telomeric DNA structure, stabilizing the T-loop and possibly limiting DNA accessibility to telomerase [26]. Conversely, TNKS1 positively regulates telomere length by the polyADP-ribosylation (PARsylation) of TRF1 thus preventing TRF1 from binding to telomeres [26,27]. Similar regulation occurs through the communication of TRF1 and POT1 [28,29]. The fact that (i) many telomere-localized and FA proteins were found among DNA repair proteins [30,31]; (ii) FANCD2 was shown to bind Holliday junction DNA, an intermediate of homologous recombination and stalled replication forks [32]; and (iii) FA proteins are involved in the ALT mechanism of telomere maintenance [33,34] prompted us to test for the possible engagement of FANCD2 in telomeres of normal cells.

Here we demonstrate that FANCD2 interacts both with telomeric DNA and TNKS1 in vitro. Moreover, FANCD2 inhibits TNKS1 PARsylation activity and TNKS1-mediated TRF1 PARsylation. Such a novel function of FANCD2 may provide an additional step for maintaining genomic stability.

## Results

### FANCD2 binds to telomeric DNA in vitro

**Figure 1:**
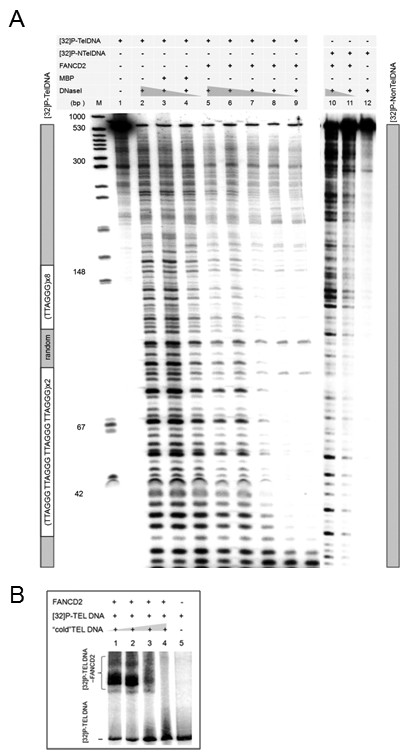
**FANCD2 binds to telomere DNA sequence**. (A) FANCD2-dependent DNase I protection assay show degradation of 32P-labeled 600 bp DNA containing either two 48 bp inserts of tandem telomeric sequences (lanes 1-9) or nonTelDNA of the same length (lanes 10-12) upon treatment with increased concentrations of DNaseI (lanes 2-11), as described in Experimental procedures. Myelin binding protein (MBP) was added as a non-FANCD2 negative control; (B) Mobility shift assay show that incubation of [32P]-labeled 40 bp telomere DNA with recombinant full length FANCD2 protein resulted in protein-DNA complex formation (lane1) identified on native gels. This complex was competed by increasing amounts of cold DNA, indicating the specificity of the telomere binding (lanes 2-4). Please note, that DNAse concentrations are comparable in lanes 3,7,10 and 4,8,11.

**Figure 2:**
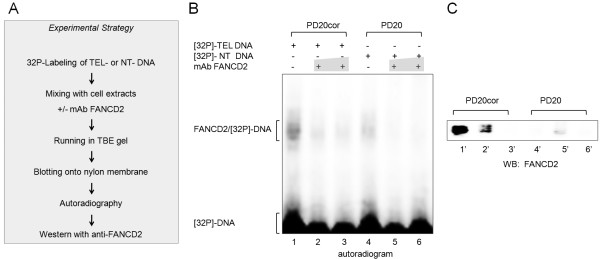
**Binding of FANCD2 and TelDNA**. (A) Experimental scheme showing specific binding of recombinant FANCD2 protein from cell extracts with TelDNA oligoduplexes. Duplex oligonucleotides containing TelDNA sequence were [32P]-end labeled and incubated with 100 μg of PD20 or PD20cor cell extracts. Complexes were separated and either subjected to autoradiography (B) or transferred to nitrocellulose for Western blot analysis with anti-FANCD2 antibody (C). The position of specific complexes formed with 32P-TelDNA and proteins from FANCD2-/- (PD20) or FANCD2-/- corrected (PD20cor) cell extracts separated on native gel matches the FANCD2 signals from the corresponding immunoblot. These specific complexes are not seen in PD20 samples.

We attempted to find a novel role for FANCD2 at telomeres and tested whether FANCD2 might bind to telomeric DNA sequence in vitro. The footprint analyses demonstrate that FANCD2 has preferential binding to a DNA template that includes tandem of 48 bp telomere sequences (Figure 1A, lanes 5-9). This binding seems to be telomere sequence-specific since adding myelin protein (Figure 1A, lanes 3,4) or using a DNA template containing nonTelDNA of the same length (Figure 1A, Lanes 10-12) revealed no FANCD2-mediated protection against DNaseI. These results were further validated by the electrophoretic mobility shift assay (EMSA). We have shown that the incubation of 40 bp telomere DNA with recombinant full length FANCD2 protein resulted in the formation of a protein-DNA complex on a native gel (Figure 1B). This complex was completed by increasing amounts of cold DNA, indicating the specificity of the telomere binding (Figure 1B, lanes 1-4). We further verified the presence of FANCD2 in DNA-protein complexes formed in EMSA by direct Western blot analysis (Figure 2) and confirmed the presence of FANCD2 in complexes formed with FANCD2 proficient (Figure 2B, lanes 1-3), but not with FANCD2 deficient cellular extracts (Figure 2B, lanes 4-6). The formation of FANCD2-32P-TelDNA complexes in native conditions was disrupted by the addition of anti-FANCD2 mAb probably due to the occlusion of FANCD2 and the fact that it competed for binding with the telomere DNA template (Figure 2B, lanes 2,3,5,6) but not with random DNA sequence (data not shown). More importantly, the mobility of 32P-TelDNA bound protein complexes on autoradiogram corresponds to the mobility of the FANCD2 signals as revealed by immunoblot analyses of the same replica gel (Figure 2C). We also demonstrated the presence of FANCD2 at telomeres in alternatively lengthening telomere (ALT) cells but not in primary cells, by performing ChIP assay (data not shown).

### FANCD2 binds to TNKS1

**Figure 3:**
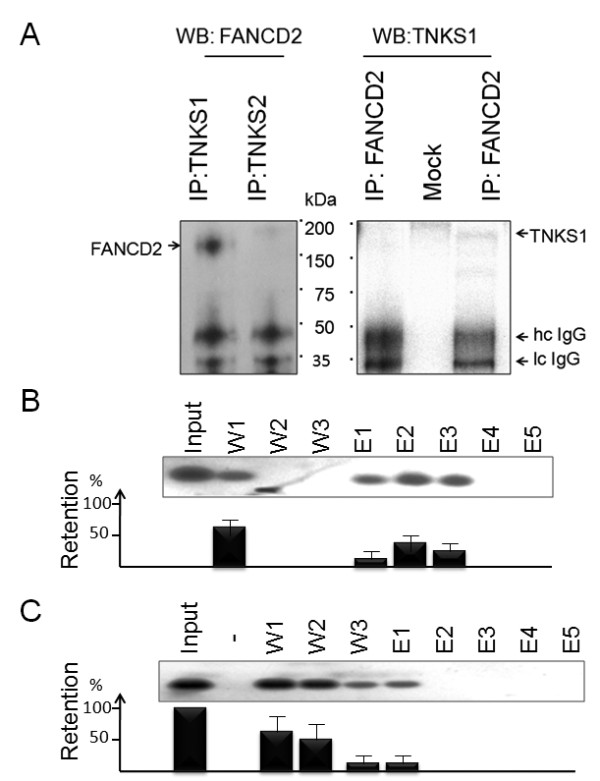
**FANCD2 binding to TNKS1**. (A) HeLa cell extracts were immunoprecipitated either with anti-tankyrase 1 (TNKS1) or anti-tankyrase 2 (TNKS2) antibody followed by Western blotting and probing with anti-FANCD2 antibodies. In parallel, same amount of cell lysate was immunoproecipitated with anti-FANCD2 antibodies and corresponding Western blot was probed with TNKS1 antibodies. Heavy and light IgG chains are depicted by arrows to show equal loading. (B) In-vitro pull-down assay showing binding of recombinant FANCD2 protein to TNKS1 was performed with 5 ug of His-tagged TNKS1 (INP, input of half TNKS1 amount used for binding) immobilized onto resin followed by incubation either with recombinant (B) or with heat-inactivated FANCD2 (C). The beads were washed with TNE buffer (fractions W1-3) followed by several elution steps (E1-5). Corresponding aliquots from each step (1/4 volume each) were subjected to Western blot analyses followed by probing with anti-FANCD2 antibodies. Densitometric measurement of the Western blot signals was performed by TotaLab2.0 software and corresponding intensities from the all fractions versus input were calculated as percentages of retained proteins.

To interpret the results of FANCD2 involvement at telomeres, we have screened some telomere-associated proteins in HeLa cells by performing immunoprecipitation experiments and detected that PARP-family member tankyrase 1 (TNKS1), but not tankyrase 2 (TNKS2), binds to FANCD2 (Figure 3A). We then confirmed, by means of an in vitro pull-down assay, that TNKS1 binds to FANCD2. For that part, His-tagged TNKS1 was immobilized on a resin followed by consecutive washing steps and binding with recombinant FANCD2 (Figure 3B) or heat-inactivated FANCD2 protein (Figure 3C). Resin complexes were washed (lanes W1-3) and eluted several times (Figure 3B, lanes E1-E5). Fractions of equal volumes were loaded onto SDS PAGE followed by Western blot analyses with anti-FANCD2. Almost 84% of FANCD2 protein (vs. 9% for heat-inactivated FANCD2) was retained with immobilized TNKS1 pellets, as calculated by comparing densitometry signals from the input to those revealed from elution fractions. The presence of single- and double-stranded telomeric and nontelomeric DNA oligonucleotides did not significantly affect this interaction (data not shown).

### FANCD2 inhibits TNKS1 and TRF1 PARsylation

**Figure 4:**
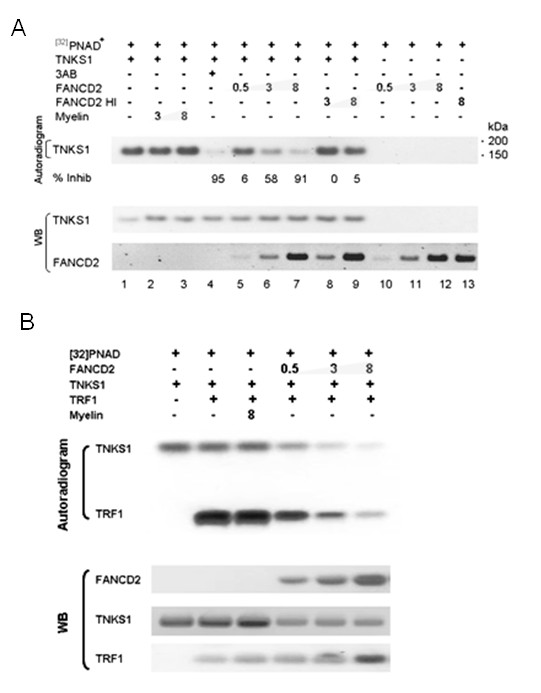
**FANCD2 inhibits tankyrase-1 PARsylation**. (A) TNKS1 (3 ug, lanes 1-9) was mixed with increasing amounts of myelin (lanes 2,3), FANCD2 (5 ug, lanes 5-7, 10-12), heat-inactivated FANCD2 (5 ug, lanes 8,9,13) in the presence of [^32^P]NAD^+ ^(lanes 1-13) and PARP inhibitor 3AB (1 mM, lane 4). After incubation in a PARP buffer, all the reactions were stopped and resulting samples were subjected to SDS-PAGE followed either by autoradiography of the membranes or detected with corresponding antibodies. as described in Experimental section; (B) FANCD2 inhibits tankyrase-1-dependent TRF1 PARsylation. TRF1 protein (3 ug) was added to the reaction mixture as in A. All the reaction products were resolved into SDS PAGE and subjected transfer into nitrocellulose membrane in the buffer containing sodium vanadate. Corresponding membranes were either autoradiographed or probed with anti-FANCD2, anti-TNKS1 or anti-TRF1 antibodies.

Using TNKS1 autoPARsylates [28], we then performed an *in vitro *PARP assay in the presence or absence of FANCD2. Interestingly, we observed the FANCD2-dependent inhibition of TNKS1 autoPARsylation (Figure 4A, lanes 5-7, upper panel). A similar effect was observed when using the PARP inhibitor 3AB (lane 4). As a control, we showed that neither myelin (lanes 2,3) or heat-inactivated FANCD2 protein (lanes 8,9) affected TNKS1 autoPARsylation. Similarly, we recognized low levels of PARsylation in TNKS1 immunocomplexes obtained after the immunoprecipitation of FANCD2-deficient PD20 cell lysates as compared with the lysates from the PD20 cells corrected with non-ubiquitinable mutant (K561R) or wild-type FANCD2(data not shown) suggesting that FANCD2-TNKS1 interaction and inhibition of TNKS1 autoPARsylation is not dependent on FANCD2 monoubiquitinylation.

Since the main known cellular function of TNKS1 is PARsylation of the telomere repeat binding factor TRF1 that results in detaching TRF1 from telomere complexes [26,27], we performed an in vitro TRF1/TNKS1 PARP assay in the presence of recombinant FANCD2. Similar to the above data, we observed the FANCD2-dependent inhibition of TRF1 PARsylation (Figure 4B). The presence of double-stranded telomeric DNA only slightly increased the inhibition of TRF1 PARsylation (data not shown). Thus, our results suggest that FANCD2 inhibits the autoPARsylation of TNKS1 and PARsylation of TRF1 in vitro.

### FANCD2 affects TRF1 binding to telomeric DNA

**Figure 5:**
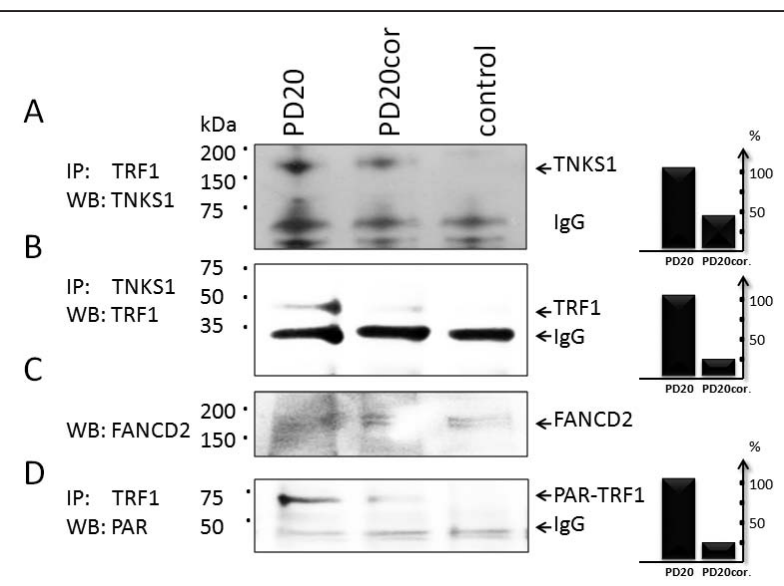
**Presence of FANCD2 rescues TRF1 from PARsylation by impairing TNKS1-TRF1 complex formation**. (A) Immunoprecipitation of PD20 or PD20cor cell extracts with TRF1 (A,D) or TNKS1(B) antibodies or non-immune control (β-actin) were performed as in Materials and Methods and corresponding immunopellets were resolved on 4-12%SDS PAGE followed by Western blotting and probing with TNKS1 (A) or TRF1 antibodies (B). FANCD2 levels in the same cell extracts is shown on Western blot (C). In parallel, immunoblots with TRF1 immunopellets were probed with anti-PAR antibodies (D). Corresponding signals of TNKS1, TRF1 or PAR-TRF1 proteins were digitized from the scanned blots and the relative percentages were included on the right side of each immunoblot panel as bar diagrams.

Previous results showed that the ADP-ribosylation of TRF1 by TNKS1 releases TRF1 from telomeres and promotes telomere elongation [27]. PARsylated TRF1 dissociates from telomeres and becomes degraded via the proteasomal pathway [35]. Deprotected telomeres become better substrates for telomerase-mediated DNA extension [36]. Since FANCD2 inhibits TNKS1 activity in vitro, we tested whether TRF1 PARsylation or TNKS1-TRF1 complex formation is affected by FANCD2. We performed multiple immunoprecipitation studies and showed that the formation of the TRF1-TNKS1 complex is impaired in FANCD2-/- cells (Figure 5A,B). This could be due to inhibition of the TNKS1-dependent degradation of TRF1. In order to test these hypotheses, we immunoprecipitated TRF1 in the above cell lysates and probed the corresponding immunoblot with anti-PAR antibodies. We recognized that in immunopellets obtained from PD20 (FANCD2-/-) cell extracts, TRF1 is more PARsylated when compared to PD20 corrected cells, suggesting that the presence of FANCD2 impairs TRF1 PARsylation (Figure 5D).

**Figure 6:**
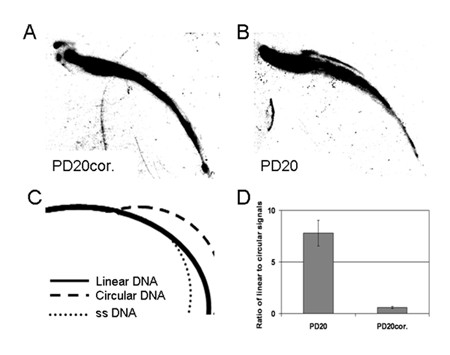
**FANCD2 deficiency leads to formation of extrachromosomal telomeric structures**. 2D Southern blot analyses of telomeric DNA signals of PD20 (A) and PD20 corrected cells (B). Scheme representing different types of extrachromosomal DNA resolved on two dimensional gel electrophoresis (C). Densitometric analyses of linear and circular extrachromosomal telomeric structures (D).

Since both the FANCD2-mediated inhibition of TNKS1 and TRF1 PARsylation may disturb TRF1 binding to telomeres, we hypothesized that FANCD2 deficient cells might have altered telomere structure, length or predisposition to recombination. Although telomere length was not affected by FANCD2 deficiency as measured by Southern blot (additional file 1), Q-FISH (additional file 2), or Flow-FISH (additional file 3) analyses, we observed telomeric extrachromosomal circular structures (ECS) in PD20 cells, but not in the corrected counterparts, by performing 2D Southern blot with a telomeric probe (Figure 6). We then attempted to compare telomere recombination in these cells and analyzed telomeric sister chromatid exchanges (T-SCE) using the CO-FISH[0] technique with double color telomeric PNA probes in PD20, PD20 corrected, primary FA-D2 and corrected cells. We did not find any statistically significant differences (additional file 4) in the amount of T-SCE between FANCD2 deficient or corrected cells indicating that the occurrence of ECS is unrelated to telomeric recombination between sister chromatids.

## Discussion

While normal FANCD2 activity is evident for maintaining living functions of any cell, it is yet unclear whether the key FA protein FANCD2 may possess biochemical properties other than crosstalk with the FA members upon resolving stalled replication forks. Our current study suggests that FANCD2 may serve a separate role, separate to FA-complex-mediated FANCD2/FANCI monoubiquitinylation. This role includes the inhibition of tankyrase 1-dependent TRF1 PARsylation which, in turn, protects telomeric DNA. We suggest that under normal conditions, FANCD2 safeguards genomic integrity both by inhibiting TNKS1-mediated TRF1 PARsylation and by protecting telomeric DNA through TRF1. TRF1 binding to telomeric DNA is essential for telomere function, whereas PARsylation of TRF1 reduces TRF1 affinity for telomeric DNA.

Recent studies showed that overexpression of TNKS1 in normal human cells results in the downregulation of TRF1, but has no effect on telomere length [37]. In addition, inhibition of TRF1 in normal human (IMR90) cells using TRF1-dominant negative allele had no effect on telomere length [31]. It was reported previously that the knockdown of FANCD2 rapidly causes telomere dysfunction in cells that rely on ALT mechanism [33]. Studies by Fan *et al. *suggest telomeric localization of FANCD2 in ALT cells and that the transient depletion of FANCD2 or FANCA results in a loss of detectable telomeres in ALT, but not in telomerase-positive cells [34]. Some previous works on telomerase-mediated telomere lengthening and generation of ECS by telomere trimming upon inhibition of TRF1 [38] incited us to examine possible changes in telomere length in our model. However, measurements of telomere length in siRNA FANCD2 depleted primary fibroblasts either using Southern blot analyses of genomic DNA (additional file 1), Q-FISH analyses in cells' metaphase (additional file 2), or Flow-FISH technique in interphase (additional file 3) did not reveal any significant changes. We can not disregard the fact that this short period of the transient depletion of FANCD2 by siRNA may not be sufficient to detect alterations in telomere length. Moreover, several other studies demonstrated telomere attrition in the FA cells of an upstream FA subtype [11,39-41]. For instance, telomere length was shown to be shorter in 54 FA patient samples, compared to 51 controls [39]. Similarly, a correlation between severe aplastic anemia and the individual annual telomere-shortening rate in peripheral blood mononuclear cells was observed in 71 FA patients [42]. These facts may suggest that telomere shortening may occur in some upstream FA subgroups as a consequence of proliferative stress. The role of FANCD2 binding with TNKS1 and telomeric DNA is still quite intriguing. On the one hand, such binding may affect telomere maintenance by protecting telomeric DNA, in a similar manner to the role of shelterin. On the other hand, FANCD2 functions as a modulator of TNKS1 activity which, in turn, may affect telomere maintenance.

Based on our experiments aimed at justifying the direct link of FANCD2 to changes in telomere length we may conclude that the presence of FANCD2 may only affect telomere stability, but not the length. Moreover, our studies on a variety of human cells do not exclude that FANCD2-dependent telomere alteration also involves other aspects of FANCD2 function. Those aspects could include the role of FANCD2 at replication fork or the recently proposed link between FANCD2 and oxidative damage [43]. Consequently, the TRF1 removal described here can be one of the possible effects of FANCD2 in telomere biology. Another interpretation could be that FANCD2 stabilizes the t-loop structure. Although our data do not demonstrate this possibility, they are highly suggestive due to the following reasons: (i) homologous recombination at t-loop leads to an extra-chromosomal circle in the presence of TRF2 deltaB [4]; (ii) FANCD2 binds Holliday junction, which might contribute to t-loop stabilization [32,44], (Giraud-Panis and Gilson, personal communication); (iii) TRF1 contributes to t-loop formation [31]. Overall, our finding of a novel interaction of FANCD2 may contribute to understanding the differences between downstream FA-D2 versus upstream FA groups.

## Conclusions

FANCD2 interacts both with telomeric DNA and with telomeric protein TNKS1 in vitro. Moreover, FANCD2 inhibits TNKS1 autoPARsylation and TNKS1-dependent TRF1 PARsylation thus affecting stability but not the length of telomeres. This novel interaction of FANCD2 may provide an additional safeguard role to secure genome integrity.

## Methods

### Cell Lines and Treatment

The following cell lines were used in this study: human transformed fibroblasts PD20 (FANCD2-/-); PD20 cor. (PD20 retrovirally corrected with pMMp-FANCD2 cDNA); PD20 transduced with pMMp-FANCD2/K561R MRC5; HeLa and primary fibroblasts (yoli) All the cell lines were cultured as described previously [45]. Importantly, PD20 cells and variants are SV40 transformed fibroblast and they are telomerase positive, as studied by both RT-PCR of hTETR and with the TRAP assay. 3AB PARP inhibitor (Sigma) was used at 3 mM concentration. Treatment with PARP inhibitor at this concentration did not significantly affect the cell cycle, as measured by flow cytometry (data not shown). The cell cycle was verified by flow cytometry and then analyzed using BD PharMingen FACScan and CellQuest software exactly as described earlier [45].

### Preparation of Human Cell Lysates

Cells were washed with ice-cold PBS and resuspended in the buffer consisting of 20 mM HEPES (pH 7.9), 420 mM KCl, 25% glycerol, 0.1 mM EDTA, 5 mM MgCl2, 0.2% Nonidet P-40, 1 mM dithiothreitol, and a 1:40 volume of protease and phosphatase inhibitor mixture (Sigma) on ice for 30 min. After centrifugation at 12,000 g for 10 min at 4°C, the supernatant was collected as the total cell lysate. Separation on nuclear and cytoplasmic fractions has been performed as described in Bogliolo *et al*. [45]. PBS-washed nuclear cell pellets were further resuspended in buffer containing 5 mM MgCl2/PBS, 10 mM HEPES (pH 7.9), 10 mM KCl, 20% glycerol, 1 mM dithiothreitol, and a protease inhibitor mixture (Sigma). The pellets were homogenized on ice by sonication, clarified by centrifugation and stored at -20°C for further experiments.

### DNA Templates and Foot-Print analyses

Telomere DNA for footprint analysis was constructed by modifying telomere sequences obtained from Dr. Giraud-Panis [44] by cutting out the right-size fragment from puc19-based pTelo2 and amplifying it by PCR following digestion with TspRI, gel purification and subsequent ligation with a non-telomeric coding 31-mere DNA linker. After subcloning to pML20-42 plasmid, 600 bp DNA strands contains two telomere tandem repeats of 48 bp each: (TTAGGG)8AACATCACGTACGTACGTACGTTCAAGCACT(TTAGGG)8 and non-telomere linker. The complete templates were gel purified and used for assay. To obtain the random-sequence of non-telomeric DNA templates, two single-stranded DNA fragments were synthesized and subcloned to pML20-42. One DNA fragment was [32P]- 5' end-labeled with T4 kinase. After annealing, the fragments were extended with Klenow(exo-) DNA polymerase and PCR amplified, followed by digestion with TspRI restriction enzyme and purified. To obtain the nonTelDNA for binding assays, same-length plasmid having nonTelDNA random sequence was used. For in-vitro pull-down assay, (TTAGGG)7 templates were synthesized and used either as single stranded DNA or annealed with corresponding sequences to obtain double stranded DNA. Consequently, ssDNA and dsDNA oligonucleotides of the same length with nonTelDNA random sequences have been utilized as controls. Foot-print analyses were performed as described in earlier study [46].

### Preparation of Proteins

Recombinant full-length wild-type FANCD2 was expressed using baculovirus in Sf9 insect. The encoded FA protein contained at the amino terminus a zz domain, for purification on IgG-Sepharose. Tagged genes were cloned into a modified pFastBac vector (Invitrogen) and processed to generate recombinant baculovirus and used to infect Sf9 cells following purification and TEV cleavage procedure exactly as described earlier [32]. TRF1 protein obtained from Dr. Chapman (MRC Laboratory of Molecular Biology, Cambridge, UK) was produced from hTRF1 expressed in E. coli following necessary purification steps. Recombinant tankyrase protein obtained from Dr. Smith was purified from the baculovirus-derived DH10Bac E. coli plasmid designed as a NH2-terminally (His)_6_-tagged version of human tankyrase in the expression vector pFastBac HTb (Gibco BRL) following expression in SF9 insect cells exactly as described before [28].

### Electrophoretic mobility shift assay (EMSA)

EMSA was performed as described in Promega protocol (http://www.promega.com/guides/protein.interactions_guide/chap7.pdf). Briefly, aliquots of FANCD2 recombinant protein or cell extracts were incubated with the DNA sequence that has been P32-labeled and the DNA:protein complexes, with or without competitors (protein or specific antibody) were run on a non-denaturing polyacrylamide gel. After electrophoresis, the experimental reaction was compared to a control reaction that contains only the labeled DNA or to the signals of corresponding immunoblot done in parallel.

### Immunoprecipitation and Western blot

For Western blot procedure cell lysates were eluted by boiling in SDS gel sample buffer and proteins were separated by SDS-PAGE and immunoblotted onto nylon membrane following incubation with corresponding antibodies. Protein bands were visualized with anti-rabbit or anti-mouse IgG coupled to horseradish peroxidase using the ECL kit (Amersham, Arlington Heights, IL, USA).

### In vitro binding and pull-down assay

For his pull-down assays, 5 ug of tankyrase 1 fusion protein was incubated for 1 h at 4°C with Co-sepharose (Pharmacia) in TNE buffer (10 mM Tris-HCl [pH 7.8], 1% Nonidet P-40 [NP-40], 150 mM NaCl, and 1 mM EDTA). Recombinant or heat inactivated (HI) FANCD2 (both at 5 ug) was added to the binding mixture either alone or in the presence of single- or double-stranded 42 bp DNA oligonucleotides following incubation in TNE buffer for 30 min at 4°C. The beads were washed with TNE buffer 3 times and corresponding aliquots were subjected to Western blot analyses as described above (fractions W1-3). Elution was done with immidazole (0.01-0.5 M). Corresponding fractions (E1-5) were collected and analysed by Western blot as above. For measurement of protein retention, scanned Western blots were analyzed by densitometry program (Totalab2.0) and the percentage of retained proteins in elution fractions was calculated by comparing the signals from input fractions (2.5 ug of each protein).

### PARP assay

Modified PARP activity assays were done with baculovirus-derived TNKS1 essentially as described before [28,47]. Shortly, (His)6-tagged version of human tankyrase samples (3 ug) and/or FANCD2 (0, 0.5, 3 or 8 ug) were incubated for 30 min at 25°C in PARP buffer 50 ul of buffer containing 50 mM tris-HCl (pH 8.0), 4 mM MgCl2, 0.2 mM dithiothreitol (DTT), 1.3. M [32P]NAD+ (4 uCi). Reactions were stopped by addition of 20% trichloroacetic acid (TCA). Acid-insoluble proteins were collected by centrifugation, rinsed in 5% TCA, suspended in Laemmli loading buffer, and fractionated by SDS-polyacrylamide gel electrophoresis (4-20% SDS-PAGE). Proteins were visualized by Western blotting with corresponding antibodies and/or autoradiography of the membranes. As controls, myeling or heat-inactivated FANCD2 (obtained by boiling FANCD2 samples for 20 min) were used. PARP inhibitor 3AB was added where needed.

## Competing interests

The authors declare that they have no competing interests.

## Authors' contributions

Authors' contributions: Please indicate that all authors read and approved the final manuscript. AL designed the work, performed experiments and wrote the manuscript. MJS, AC, MC, AMS performed experiments and interpreted the data. JP interpreted the data and participated in manuscript drafting. JS interpreted the data, designed the experimental strategy, participated in manuscript drafting and obtained funds for making this study.

## Supplementary Material

Additional file 1 **Southern blot analyses of telomere length upon FANCD2 depletion**. (A) Primary fibroblasts (PR) were consecutively depleted from FANCD2 by siRNA for 5 days (PR+siRNA) or treated with scrambled RNA (PR+scRNA) and the telomere length was measured by Southern Blot analyses probed with Telomere probe as described in the manufacturer manual from Roche. Low (Tel Con Low), high (Tel Con High) control telomeric DNAs (Roche) or DNA extracted from mouse fibroblast (Tel Con M) or HeLa cells at 2 ug or 5 ug were applied alongside with the PR probes. (B) Telomere length was measured by computer program (Roche) as the mean of the maximum intensities. Click here for file

Additional file 2 **Q-FISH analyses of telomere length upon FANCD2 depletion**. Human primary fibroblasts (control) were consecutively depleted from FANCD2 by siRNA for 5 days (FANCD2 siRNA) or treated with scrambled RNA (scRNA) and the telomere length was measured by Q-FISH analyses as described in Slijepcevic [48]. Mann-Whitney test was applied to interpret the significance of the data and corresponding P values are depicted at the bottom of the figures. Click here for file

Additional file 3 **Flow-FISH analyses of telomere length upon FANCD2 depletion**. Human primary fibroblasts (control) were consecutively depleted from FANCD2 by siRNA for 5 days (FANCD2 siRNA) or treated with scrambled RNA (scRNA) and the level of FANCD2 was tested either by Western blot (A) or by immunofluorescence (B, green signals). Corresponding fractions were measured for telomere length by Flow-FISH analyses as described in Baerlocher et al.[49]. Click here for file

Additional file 4 **Telomere recombination in FANCD2 deficient cells**. FANCD2-/- or FANCD2-/- corrected transformed (left part) or primary (right part) fibroblasts were assayed for telomeric sister chromatid exchanges (T-SCE) and the relative number of T-SCE/chromosome was were measured by CO-FISH[0] technique with double color telomeric PNA probes followed by plotting on the diagram. Click here for file
