# Supplementary figures and images for: Fanconi anemia protein FANCD2 inhibits TRF1 polyADP-ribosylation through tankyrase1-dependent manner
Source: Genome Integr. 2011 Feb 12;2:4. doi: 10.1186/2041-9414-2-4 (PMC3048478; doi:10.1186/2041-9414-2-4)

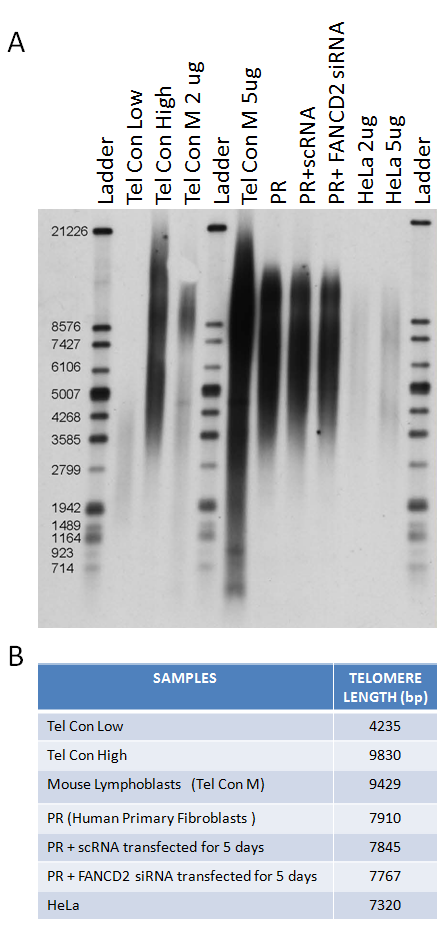

Supplement: Additional file 1 — Southern blot analyses of telomere length upon FANCD2 depletion. (A) Primary fibroblasts (PR) were consecutively depleted from FANCD2 by siRNA for 5 days (PR+siRNA) or treated with scrambled RNA (PR+scRNA) and the telomere length was measured by Southern Blot analyses probed with Telomere probe as described in the manufacturer manual from Roche. Low (Tel Con Low), high (Tel Con High) control telomeric DNAs (Roche) or DNA extracted from mouse fibroblast (Tel Con M) or HeLa cells at 2 ug or 5 ug were applied alongside with the PR probes. (B) Telomere length was measured by computer program (Roche) as the mean of the maximum intensities. [file 2041-9414-2-4-S1.TIFF]

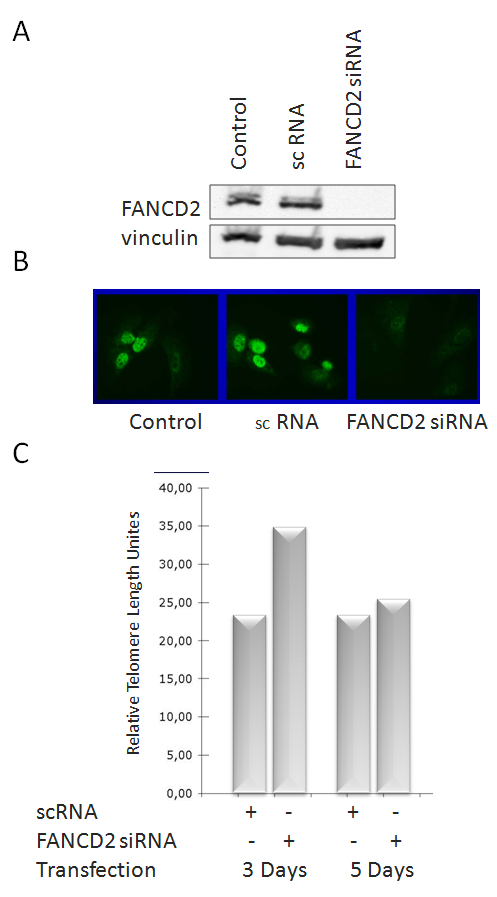

Supplement: Additional file 2 — Q-FISH analyses of telomere length upon FANCD2 depletion. Human primary fibroblasts (control) were consecutively depleted from FANCD2 by siRNA for 5 days (FANCD2 siRNA) or treated with scrambled RNA (scRNA) and the telomere length was measured by Q-FISH analyses as described in Slijepcevic [48]. Mann-Whitney test was applied to interpret the significance of the data and corresponding P values are depicted at the bottom of the figures. [file 2041-9414-2-4-S2.TIFF]

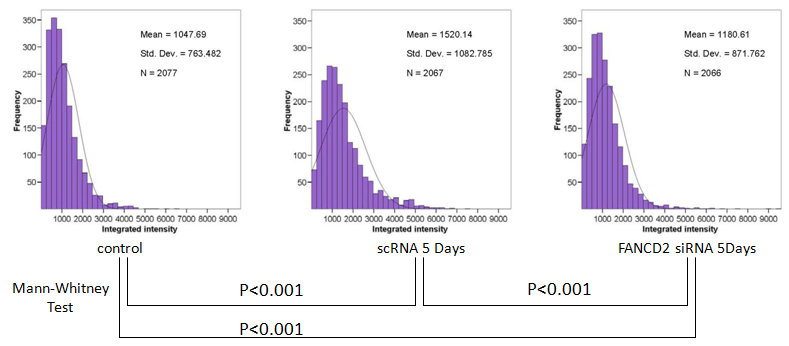

Supplement: Additional file 3 — Flow-FISH analyses of telomere length upon FANCD2 depletion. Human primary fibroblasts (control) were consecutively depleted from FANCD2 by siRNA for 5 days (FANCD2 siRNA) or treated with scrambled RNA (scRNA) and the level of FANCD2 was tested either by Western blot (A) or by immunofluorescence (B, green signals). Corresponding fractions were measured for telomere length by Flow-FISH analyses as described in Baerlocher et al.[49]. [file 2041-9414-2-4-S3.TIFF]

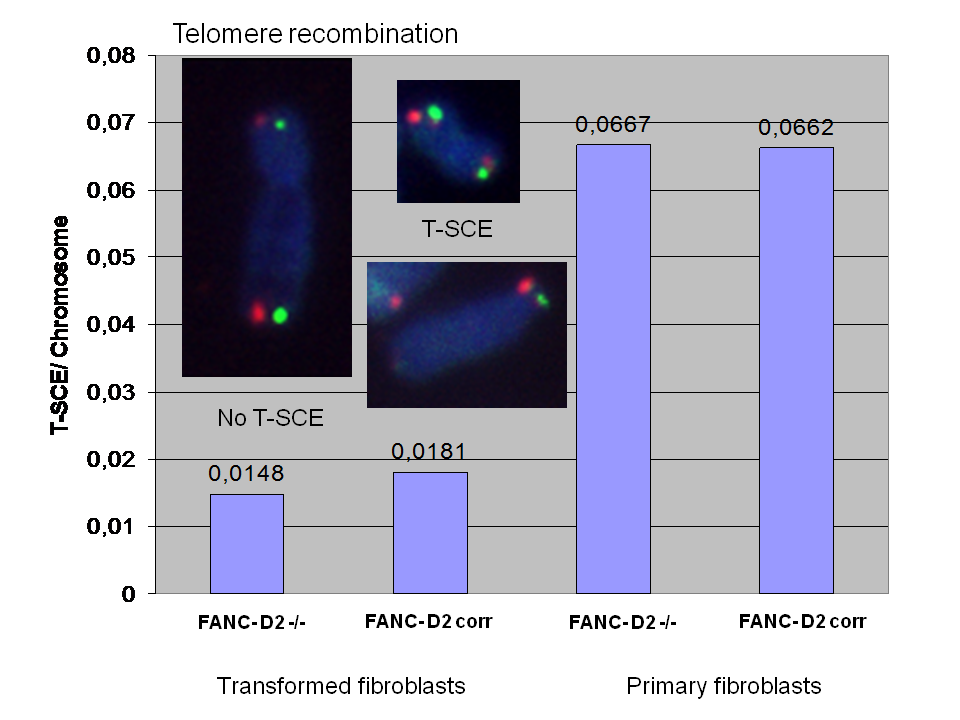

Supplement: Additional file 4 — Telomere recombination in FANCD2 deficient cells. FANCD2-/- or FANCD2-/- corrected transformed (left part) or primary (right part) fibroblasts were assayed for telomeric sister chromatid exchanges (T-SCE) and the relative number of T-SCE/chromosome was were measured by CO-FISH[0] technique with double color telomeric PNA probes followed by plotting on the diagram. [file 2041-9414-2-4-S4.TIFF]
